# NAD^+^ supplementation augments the efficacy of the PARP1 inhibitor PJ34 in a 6-OHDA-induced model of Parkinson’s disease

**DOI:** 10.1016/j.gendis.2025.101783

**Published:** 2025-07-25

**Authors:** Mengling Hu, Xiaoqian Li, Dongsheng Fan, Lu Yu, Fan Ren, Jianming Wu, Jianing Mi, Yang Zheng, Xiaogang Zhou, Dalian Qin, Anguo Wu

**Affiliations:** a Department of Pharmacy, Guang’an People’s Hospital, Sichuan 638550, China; b State Key Laboratory of Traditional Chinese Medicine Syndrome, The Second Affiliated Hospital of Guangzhou University of Chinese Medicine, Guangzhou, Guangdong 510120, China; c Sichuan Key Medical Laboratory of New Drug Discovery and Drugability Evaluation, Key Laboratory of Medical Electrophysiology of Ministry of Education, Institute of Drug Clinical Trial/GCP Center, Department of Cardiology of The Affiliated Hospital of Southwest Medical University, School of Pharmacy, Southwest Medical University, Luzhou, Sichuan 646000, China; d Department of Pharmacy, First Affiliated Hospital, Guizhou University of Traditional Chinese Medicine, Guiyang, Guizhou 550000, China; e Chongqing Key Laboratory of Sichuan-Chongqing Co-construction for Diagnosis and Treatment of Infectious Diseases Integrated Traditional Chinese and Western Medicine, Chongqing Traditional Chinese Medicine Hospital, Chongqing 400021, China

Parkinson’s disease (PD) is a neurodegenerative disorder characterized by dopaminergic neuronal loss, mitochondrial dysfunction, and neuroinflammation.^1^ Hyperactivation of poly(ADP-ribose) polymerase 1 (PARP1) contributes to PD pathology by depleting nicotinamide adenine dinucleotide (NAD^+^) and promoting cell death. While PARP1 inhibitors like PJ34 can attenuate neurodegeneration, their efficacy may be limited when used alone.^2^ NAD^+^ supplementation has shown promise in maintaining mitochondrial integrity and reducing inflammation, but has not been extensively combined with PARP1 inhibition.^3^ This study explores the therapeutic synergy of co-administering NAD^+^ and PJ34 in a 6-hydroxydopamine (6-OHDA)-induced PD model.^4^ We demonstrate that this combination enhances neuronal survival, suppresses neuroinflammation, and promotes autophagic flux more effectively than monotherapies, providing a compelling strategy to concurrently target energy metabolism and DNA repair pathways.

Oxidative stress is a key mechanism in PD, leading to DNA damage and excessive activation of PARP1.^2^ In this study, we used H_2_O_2_ to induce oxidative stress in SH-SY5Y cells. H_2_O_2_ treatment at 0.5–2 mM resulted in a dose-dependent increase in PAR expression (Fig. S1A–S1C). Both NAD^+^ and PJ34, at non-toxic concentrations, showed protective effects by enhancing cell viability and reducing cell death, with the combination treatment demonstrating significantly superior efficacy compared with either treatment alone (Fig. S1D–S1M). Immunofluorescence analysis further revealed that the combination treatment significantly reduced PAR expression, DNA damage markers, including phosphorylated histone variant H2A.X (γH2A.X) and tumor suppressor p53-binding protein 1 (53BP1), and reactive oxygen species levels (Fig. S1K, S1M–S1O). These results indicate a synergistic effect of NAD^+^ and PJ34 in attenuating H_2_O_2_-induced cytotoxicity, suggesting enhanced neuroprotective potential through their combined use.

To evaluate the neuroprotective effects of NAD^+^ and PJ34 against neurotoxicity induced by PD neurotoxins, SH-SY5Y cells were treated with MPTP (short for 1-methyl-4-phenyl-1,2,3,6-tetrahydropyridine), 6-OHDA, and rotenone. Immunofluorescence and western blotting analyses revealed increased PAR expression in SH-SY5Y cells, with 6-OHDA inducing the most pronounced effect (Fig. S2A–S2D). PJ34 significantly improved cell viability and reduced cell death and DNA damage markers (γH2A.X and 53BP1) in 6-OHDA-treated cells compared with other neurotoxins (Fig. S2E–S2G). Further analysis of NAD^+^ and PJ34 in 6-OHDA-treated cells showed that both agents independently increased viability and decreased PAR and γH2A.X/53BP1 levels, while their combination offered greater protection against cytotoxicity (Fig. S2H–S2O; Fig. 1A and B). To address the multifaceted pathology of PD, we investigated whether the combination of NAD^+^ and PJ34 could enhance neuroprotective effects by targeting autophagy, mitochondrial dysfunction, cellular senescence, and inflammation. In RFP-GFP-LC3 U87 cells, the combination treatment reduced the GFP-LC3/RFP-LC3 ratio, indicating enhanced autophagic flux (Fig. S3A and S3B). In 6-OHDA-induced SH-SY5Y cells, NAD^+^ and PJ34 significantly decreased p62 accumulation, increased the LC3-II/LC3-I ratio, and improved lysosomal function, as evidenced by an increased ratio of pH-Lys Green to LysoPrime Deep Red (Fig. 1C–E; Fig. S3C and S3D). In 6-OHDA-induced BV-2 cells, the combination of NAD^+^ and PJ34 significantly suppressed nucleotide-binding and oligomerization domain (NOD)-like receptor 3 (NLRP3) inflammasome activation, reducing levels of NLRP3, caspase-1, ASC (short for apoptosis-associated speck-like protein with a caspase recruitment domain), gasdermin D (GSDMD), and pro-inflammatory cytokines interleukin-1beta (IL-1β) and IL-18, as confirmed by immunofluorescence and western blotting analysis (Fig. 1C, F–I; Fig. S4). Furthermore, cellular senescence was significantly attenuated in both bleomycin-treated A549 cells and 6-OHDA-treated SH-SY5Y cells, as shown by reduced senescence-associated β-galactosidase (SA-β-gal) staining and SPiDER-βGal fluorescence intensity after combination treatment (Fig. S5A–S5C; Fig. 1J and K). Mitochondrial integrity was also restored in 6-OHDA-treated SH-SY5Y cells, with reduced mitochondrial reactive oxygen species levels, increased mitochondrial length, and improved mitochondrial membrane potential after combination treatment (Fig. 1J, L, M; Fig. S6). Collectively, these findings suggest that NAD^+^ supplementation enhances the neuroprotective efficacy of PJ34, offering a potential strategy to mitigate oxidative stress, mitochondrial dysfunction, and inflammation in PD.

**Figure 1:**
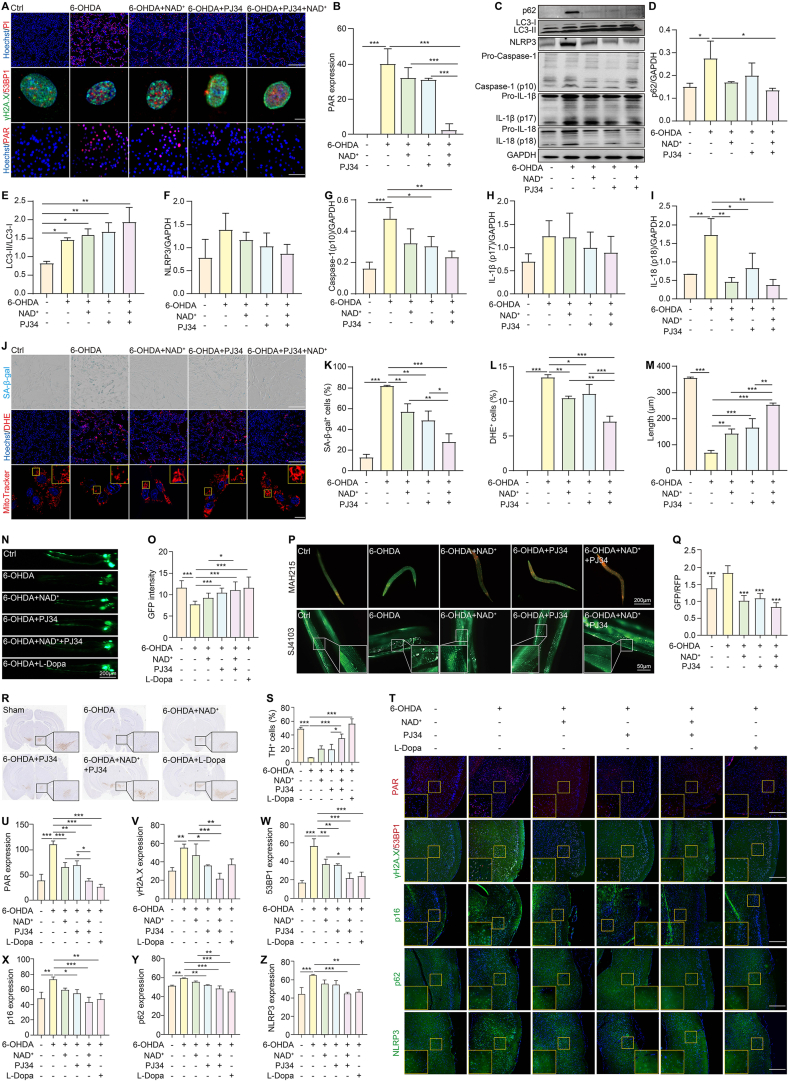
Enhancement of PJ34 efficacy by NAD^+^ supplementation in 6-OHDA-induced PD models. **(A)** Representative fluorescence images of Hoechst/propidium iodide staining, γH2A.X/53BP1 expression, and PAR expression in 6-OHDA-induced SH-SY5Y cells treated with NAD^+^, PJ34, or their combination. Magnification: 10×, 40×, or 63×. Scale bar: 200 μm, 50 μm, or 32 μm. **(B)** Quantification of the PAR expression in SH-SY5Y cells (*n* = 3). **(C–I)** Western blotting analysis and corresponding quantification of p62, LC3, NLRP3, caspase-1, IL-1β, and IL-18 in 6-OHDA-induced BV-2 cells treated with NAD^+^, PJ34, or their combination (*n* = 3). Original and non-processed western blotting images are provided in Figure S11. **(J)** Representative images of senescence-associated β-galactosidase (SA-β-gal), dihydroethidium (DHE), and MitoTracker staining in 6-OHDA-induced SH-SY5Y cells treated with NAD^+^, PJ34, or their combination. Zoomed-in images highlight the mitochondrial structure. Magnification: 10× or 63×. Scale bar = 200 μm or 32 μm. **(K**–**M)** Quantification of SA-β-gal-positive cells (%), DHE^+^ cells (%), and mitochondrial length (μm) (*n* = 3). **(N)** Representative fluorescence images of 6-OHDA-treated BZ555 worms treated with NAD^+^, PJ34, or their combination. Magnification: 20×. Scale bar: 100 μm. **(O)** Quantification of the GFP intensity in 6-OHDA-induced BZ555 worms (*n* = 20). **(P)** Representative images of GFP::LGG-1 and RFP::LGG-1 puncta in 6-OHDA-induced MAH215 worms and mitochondrial structure in the muscles of 6-OHDA-induced SJ4103 worms treated with NAD^+^, PJ34, or their combination. Zoomed-in images highlight mitochondrial structure. Magnification: 10× or 100×. Scale bar: 200 μm or 20 μm. **(Q)** Quantification of the GFP/RFP ratio (*n* = 20). **(R)** Immunohistochemical staining of tyrosine hydroxylase (TH) in the substantia nigra (SNc) of mice. Magnification: 20×. Scale bar: 100 μm. **(S)** Quantification of TH expression (*n* = 3). **(T)** Immunofluorescence images of PAR, γH2A.X/53BP1, p16, p62, and NLRP3 expression in the brain tissue, with zoomed-in sections highlighting differences. Magnification: 40×. Scale bar: 50 μm. **(U**–**Z)** Quantification of PAR, γH2A.X, 53BP1, p16, p62, and NLRP3 expression (*n* = 3). Data were analyzed by a one-way ANOVA, followed by Dunnett’s multiple comparisons test. *∗P* < 0.05, *∗∗P* < 0.01, and *∗∗∗P* < 0.001.

*Caenorhabditis elegans* (*C. elegans*) is a valuable model for studying PD due to its well-characterized dopaminergic nervous system, conserved pathways relevant to neurodegeneration (*e.g.*, mitochondrial dynamics, autophagy, oxidative stress), genetic tractability, and suitability for high-throughput phenotypic analysis.^5^ Here, we used *C. elegans* to evaluate the neuroprotective effects of NAD^+^ and PJ34 in 6-OHDA-induced models. Initially, the potential anti-aging effects of this combination were investigated in N2 worms. Compared with NAD^+^ or PJ34 alone, the combination showed significantly superior efficacy in improving body length, width, body bends, and pumping rates, as well as reducing lipofuscin levels, an indicator of oxidative stress and aging (Fig. S7A–S7G). In 6-OHDA-induced BZ555 worms, the combination treatment significantly increased GFP expression, indicating improved dopaminergic neuron viability and enhanced motor functions, including body bends and the slowing rate (Fig. 1N, O; Fig. S7H and S7I). Autophagic dysfunction caused by 6-OHDA was alleviated by the combination treatment, as evidenced by reduced GFP::p62 intensity in BC12921 worms and a decreased GFP/RFP ratio in MAH215 worms, demonstrating enhanced autophagic flux (Fig. S7J and S7K; Fig. 1P and Q). Mitochondrial integrity was also preserved in SJ4103 worms treated with the combination (Fig. 1P), while NL5901 worms showed reduced GFP::α-synuclein expression and improved body bends, indicating a reduction in α-synuclein aggregation and enhanced locomotion (Fig. S7L–S7N). These findings suggest that NAD^+^ and PJ34 synergistically improve neuronal health, enhance autophagy, and support motor function in *C. elegans* with PD.

To evaluate the combined effects of NAD^+^ and PJ34 in a mammalian model, we assessed their impact on motor function, neuronal health, and key molecular mechanisms in 6-OHDA-induced mice (Fig. S8A). Behavioral analyses showed that the combination treatment significantly improved motor function, evidenced by reduced apomorphine-induced rotations and increased average swimming and suspension times compared with individual treatments (Fig. S8B–S8D). Immunohistochemical analysis revealed enhanced preservation of tyrosine hydroxylase-positive dopaminergic neurons in the substantia nigra pars compacta with the combination therapy, suggesting superior neuroprotection (Fig. 1R and S). Markers of DNA damage and cellular stress, including PAR, γH2A.X, and 53BP1, were significantly reduced in the combination treatment group, as confirmed by immunofluorescence and western blotting analysis (Fig. 1T–W; Fig. S8E). The combination treatment also attenuated cellular senescence, enhanced autophagic flux, and reduced neuroinflammation. The expression of the senescence marker p16 was significantly decreased, while autophagy markers light chain 3 (LC3) and p62 indicated improved autophagy (increased LC3 and decreased p62 levels) and elevated lysosomal-associated membrane protein 2 (Lamp2) expression, supporting enhanced lysosomal function (Fig. 1T–Y; Fig. S8F–S8H). Moreover, neuroinflammatory markers cyclooxygenase 2 (COX-2) and NLRP3 were significantly down-regulated, and activation of astrocytes and microglia, indicated by GFAP and Iba1 staining, was markedly reduced (Fig. 1T, Z; Fig. S8F, S8I–S8M). These results indicate that NAD^+^ supplementation augments the efficacy of PJ34, leading to improved motor function, neuronal preservation, reduced cellular senescence, improved autophagy, and decreased neuroinflammation in 6-OHDA-induced mice.

In summary, this study demonstrates that NAD^+^ supplementation enhances the neuroprotective efficacy of the PARP1 inhibitor PJ34 in a 6-OHDA-induced PD model by mitigating oxidative stress, preserving mitochondrial integrity, suppressing senescence, promoting autophagy, and reducing neuroinflammation across SH-SY5Y cells, C. *elegans*, and mice (Fig. S9). The observed synergy likely arises from complementary mechanisms: PJ34 inhibits PARP1-mediated NAD^+^ depletion and DNA damage signaling, while NAD^+^ replenishment restores mitochondrial and metabolic homeostasis via sirtuin 1 (SIRT1) and AMP-activated protein kinase (AMPK) pathways. Despite these promising findings, several limitations exist, including the use of an acute toxin-based model, the absence of pharmacokinetic and brain distribution data for NAD^+^ and PJ34, and limited exploration of additional pathways such as phosphatase and tensin (PTEN)-induced kinase 1 (PINK1)/Parkin mitophagy and nuclear factor kappa B (NF-κB) signaling. Potential systemic effects from long-term PARP1 inhibition or NAD^+^ elevation, such as altered immunity or peripheral metabolic shifts, also warrant investigation. Only male mice were used to reduce hormonal variability, which may limit generalizability. Future studies should include both sexes, employ chronic PD models, refine delivery strategies, and assess systemic safety to advance clinical translation of this dual-targeted therapy.

## CRediT authorship contribution statement

**Mengling Hu:** Data curation, Methodology. **Xiaoqian Li:** Methodology, Data curation. **Dongsheng Fan:** Investigation, Validation. **Lu Yu:** Investigation, Validation. **Fan Ren:** Validation, Investigation. **Jianming Wu:** Investigation. **Jianing Mi:** Investigation. **Yang Zheng:** Writing – review & editing. **Xiaogang Zhou:** Data curation. **Dalian Qin:** Supervision, Writing – original draft, Writing – review & editing, Conceptualization. **Anguo Wu:** Funding acquisition, Supervision.

## Ethics declaration

All procedures were conducted in accordance with the guidelines for the care and use of laboratory animals and were approved by the Institutional Animal Care and Use Committee (approval number: 20240515-006).

## Funding

This work was supported by the Sichuan Science and Technology Program (China) (No. 2024YFHZ0361), the Science and Technology Strategic Cooperation Programs of Luzhou Municipal People’s Government and Southwest Medical University (China) (No. 2024LZXNYDJ026), the 10.13039/501100001809Natural Science Foundation of China (No. 81903829, 82304994), the Open Project from the State Key Laboratory of Traditional Chinese Medicine Syndrome (China) (No. SKLKY2024B0006), and 10.13039/501100001809Chongqing Natural Science Foundation of China (No. CSTB2022NSCQ-MSX1560).

## Conflict of interests

The authors declared no competing interests.
