# Supplementary material for: NAD+ supplementation augments the efficacy of the PARP1 inhibitor PJ34 in a 6-OHDA-induced model of Parkinson’s disease
Source: Genes Dis. 2025 Jul 25;13(2):101783. doi: 10.1016/j.gendis.2025.101783 (PMC12666707; doi:10.1016/j.gendis.2025.101783)
Supplement: Multimedia component 1 [file mmc1.docx]

**1. Methods and materials**

*1.1. Chemicals, antibodies, and plasmids*

6-OHDA (H4381), NAD^+^ (N3014), PI (P4170), and MTT (M2003) were purchased from Sigma‒Aldrich (St. Louis, MO, USA). PJ34 (T2124) was obtained from TargetMol (Boston, MA, USA). Bleomycin (BLM, B107423), and FUDR (F0503) were obtained from Aladdin (Shanghai, China). L-DOPA (D807434) was purchased from Macklin (Shanghai, China). Tetramisole hydrochloride (S4110) was procured from Solarbio (Beijing, China). Pentobarbital was purchased from MERYER (Shanghai, China), and apomorphine (FA027401) was purchased from Panphy (Hangzhou, China). Penicillin (ST2560) was acquired from Beyotime (Shanghai, China). Hoechst 33342 (GC10939-100) was obtained from GLPBIO (Montclair, CA, USA), and Lipomaster 3000 Transfection Reagent (TL301-02) was obtained from Vazyme (Nanjing, China). Dihydroethidium (S0063) and MitoTracker Red CMXRos (C1049B) were purchased from Beyotime (Shanghai, China). The Mitochondrial Membrane Potential Assay Kit with JC-1 (M8650) was obtained from Solarbio (Beijing, China). The Lysosomal Acidic pH Detection Kit-Green/Deep Red (L268) and Cellular Senescence Detection Kit-SPiDER-βGal (SG03) were obtained from DOJINDO (Kumamoto, Japan). β-galactosidase staining kit (G1580) was obtained from Solarbio (Beijing, China). The primary antibodies used in this study included anti-PAR (4335-MC-100) from R&D Systems (Minneapolis, MN, USA), anti-LC3 (14600-1-AP), anti-p62 (18420-1-AP), anti-NLRP3 (19971-1-AP), anti-GAPDH (60004-1-Ig), anti-IL-1β (16806-1-AP), anti-COX-2 (66351-1-Ig), and anti-GFAP (16825-1-AP) from Proteintech (Rosemont, IL, USA), anti-caspase-1 (A0964) and anti-IL-18 (A16737) from ABclonal (Woburn, MA, USA), anti-TH (sc-25269), anti-53BP1 (sc-515841), and anti-Lamp2 (sc-18822) from Santa Cruz Biotechnology (Dallas, TX, USA), anti-γH2A.X (T56572S) from Abmart (Shanghai, China), anti-Iba1 (ET1705-78) from Huabio (Hangzhou, China), anti-p16 (HY-P80992) from MCE (Shanghai, China), and p62 (P23603) from ProMab (Changsha, China). The plasmids used for transfection included pLVX-H2A.X-AcGFP1 (PPL02226-4a), pLVX-mCherry-TP53BP1 (PPL03267-4b), pGFP-N1-NLRP3 (PPL00151-2a), pEGFP-N1-caspase-1 (PPL00392-2e), pmCherry-C1-ASC (PPL01752-2b), and pLVX-AcGFP1-N1-GSDMD (PPL00857-4b) were obtained from the Public Protein/Plasmid Library (Nanjing, China).

*1.2. Cell culture*

SH-SY5Y human neuroblastoma cells were obtained from the American Type Culture Collection (ATCC, Manassas, VA, USA). All cells were cultured in Dulbecco's modified Eagle’s medium (DMEM, Gibco, Waltham, MA, USA) supplemented with 10% fetal bovine serum (FBS, Gibco), 100 U/mL penicillin, and 100 µg/mL streptomycin (Beyotime, Shanghai, China). The cells were maintained at 37°C in a humidified atmosphere containing 5% CO_2_. The medium was replaced every 2-3 days, and the cells were subcultured at approximately 80-90% confluence using 0.25% trypsin-EDTA (Gibco).

*1.3. Immunofluorescence*

Immunofluorescence staining was performed to evaluate the expression and localization of key proteins in SH-SY5Y cells and mouse brain tissues.

SH-SY5Y cells: SH-SY5Y cells were seeded on glass coverslips in 24-well plates and allowed to adhere overnight. After treatment, the cells were washed three times with PBS and fixed with 4% paraformaldehyde (PFA; Biosharp; BL539A) for 15 minutes at room temperature. The cells were then permeabilized with 0.3% Triton X-100 (Beyotime; P0096) for 10 minutes and blocked with 5% bovine serum albumin (BSA; Beyotime; ST023) for 1 hour at room temperature. Primary antibodies were diluted in 1% BSA in PBS and incubated with the cells overnight at 4°C. The primary antibodies used were as follows: mouse anti-PAR (R&D Systems, 4335-MC-100, 1:250), rabbit anti-γH2A.X (Abmart, T56572S, 1:250), and mouse anti-53BP1 (Santa Cruz, sc-515841, 1:250). After washing with PBS, the cells were incubated with the appropriate fluorophore-conjugated secondary antibodies (Invitrogen, 1:1000) for 1 hour at room temperature in the dark. Coverslips were mounted onto glass slides using ProLong Gold Antifade Mountant with DAPI (Invitrogen, P36935). Images were acquired using an epifluorescence microscope (Olympus IX73) and a confocal laser scanning microscope (Zeiss LSM 980), and analyzed using ImageJ software. Quantitative analysis of fluorescence intensity was performed from 5 randomly selected fields per coverslip in 3 independent replicates.

Mouse brain tissue: Mice were deeply anesthetized with pentobarbital (50 mg/kg, intraperitoneal; MERYER) and transcardially perfused with cold PBS followed by 4% PFA in PBS. The brains were dissected and postfixed in 4% PFA overnight at 4°C. After fixation, the brains were cryoprotected in 30% sucrose (Sigma‒Aldrich, S7903) in PBS until they sank. The brains were then embedded in an OCT compound (Sakura Finetek, 4583) and sectioned at 30 µm thickness using a cryostat (Leica CM1950). The brain sections were washed three times with PBS to remove the cryoprotectant, dipped in 0.3% Triton X-100 (Beyotime; P0096) for 15 minutes, and then blocked in goat serum (Sigma‒Aldrich, G6767) for 30 minutes. After washing again with PBS, the sections were incubated with the following primary antibodies diluted in blocking solution at 4°C overnight: mouse anti-PAR (R&D Systems, 4335-MC-100, 1:250) and rabbit anti-γH2A.X (Abmart, T56572S, 1:250), mouse anti-53BP1 (Santa, sc-515841, 1:250), rabbit anti-p16 (MCE, HY-P80992, 1:250), rabbit anti-LC3 (Proteintech, 14600-1-AP, 1:250), rabbit anti-p62 (Proteintech, 18420-1-AP, 1:250), mouse anti-Lamp2 (Santa, sc-18822, 1:250), mouse anti-COX-2 (Proteintech, 66351-1-Ig, 1:250), and rabbit anti-NLRP3 (Proteintech, 19971-1-AP, 1:250) antibodies were used. After washing with PBS, the sections were incubated with the appropriate fluorophore-conjugated secondary antibodies (Invitrogen, 1:1000) for 1 hour at room temperature in the dark. The sections were then mounted on glass slides using ProLong Gold Antifade Mountant with DAPI (Invitrogen, P36935). Images were acquired using Olympus IX73 and Zeiss LSM 980 microscopes. For each group, four mice (n = 4) were analyzed, and three non-overlapping sections per brain region were imaged. Quantitative fluorescence analysis was performed using ImageJ by measuring the mean integrated density in defined regions of interest (ROIs).

*1.4. MTT assay*

SH-SY5Y cells were seeded in 96-well plates at a density of 5 × 10^3^ cells per well and incubated overnight. After treatment, 10 µL of MTT solution (5 mg/mL, Sigma‒Aldrich) was added to each well and incubated for 4 hours at 37°C. The medium was removed, and 150 µL of DMSO (Aladdin, D103273) was added to dissolve the formazan crystals. The plates were shaken for 10 minutes. The absorbance at 570 nm was measured using a microplate reader (BioTek, Winooski, VT, USA). Cell viability was calculated as a percentage of the control group ^1^. The experiments were performed in triplicate.

*1.5. Hoechst/PI staining*

SH-SY5Y cells were seeded in 24-well plates at a density of 2 × 10^4^ cells per well and allowed to adhere overnight. After treatment, the cells were washed with PBS and incubated with 10 µg/mL of Hoechst 33342 (GLPBIO) for 10 minutes at 37°C to stain the nuclei. Following Hoechst staining, the cells were washed with PBS and incubated with 5 µg/mL propidium iodide (PI) (Sigma‒Aldrich) for 5 minutes at room temperature to stain the dead cells. The cells were then washed with PBS and observed under a fluorescence microscope (Olympus IX73). Images were captured, and at least three random fields were selected for analysis. The percentage of PI-positive cells (dead cells) relative to the total number of Hoechst-stained cells (total cells) was calculated ^2^. The experiments were performed in triplicate*.*

*1.6. DHE staining*

To assess the production of reactive oxygen species (ROS) in SH-SY5Y cells, dihydroethidium (DHE) staining was performed. SH-SY5Y cells were seeded in 6-well plates at a density of 1 × 10^5^ cells per well and allowed to adhere overnight. Following treatment, the culture medium was removed, and the cells were washed twice with PBS. A working solution of DHE (Beyotime) was prepared by diluting the stock solution to a final concentration of 10 μM in serum-free DMEM. The cells were incubated with DHE working solution at 37°C for 30 minutes in the dark. After incubation, the DHE solution was removed, and the cells were washed three times with PBS to remove excess dye. The fluorescence intensity of DHE was observed using a fluorescence microscope (Olympus IX73) with excitation and emission wavelengths of 518 nm and 605 nm, respectively. Images were captured from at least three random fields per well. The fluorescence intensity, which is indicative of ROS levels, was quantified using ImageJ analysis software (NIH) ^3^. All experiments were performed in triplicate to ensure reproducibility.

*1.7. Western blot analysis*

SH-SY5Y cells, BV-2 cells and mouse brain tissue were harvested and lysed in RIPA buffer (Proteintech, PR20001) containing protease and phosphatase inhibitors (TargetMol, S0160090). The lysates were mixed with SDS loading buffer and denatured by heating at 100°C for 10 minutes. Each protein sample was separated by SDS‒PAGE (10% gel) and transferred onto polyvinylidene difluoride membranes (PALL). The membranes were blocked with 5% (w/v) nonfat milk in TBST for 1 hour at room temperature. The membranes were then incubated with primary antibodies overnight at 4°C. After extensive washing with TBST, the membranes were incubated with HRP-conjugated secondary antibodies (anti-rabbit IgG, anti-mouse IgG; Proteintech, 1:1000) in 1% (w/v) nonfat milk in TBST for 1 hour at room temperature. Protein-antibody immunoreactivity was detected by using an enhanced chemiluminescence (ECL) detection kit (Proteintech) and imaged on a ChemiDoc imaging system (Tanon). Densitometric analysis was performed using ImageJ software.

*1.8.* β*-Galactosidase staining*

A549 cells were seeded in 6-well plates and treated with BLM (5 μg/mL), NAD^+^ (1 mM), PJ34 (5 μM), or their combination for 24 hours. SH-SY5Y cells were seeded in 6-well plates and treated with 6-OHDA (100 μM), NAD^+^ (1 mM), PJ34 (5 μM), or their combination for 24 hours. After treatment, the cells were washed twice with PBS, fixed in PBS (pH 6.0) containing 0.5% glutaraldehyde for 15 minutes, and then washed with PBS. The cells were then incubated in phosphate buffer at pH 6.0 containing 5 mM potassium ferrocyanide, 5 mM potassium ferricyanide, 1 mM MgCl_2_, and 1 mg/mL X-Gal (Beyotime, G1580) for 24 hours at 37°C. The stained cells were washed in PBS, mounted in 90% glycerol, and then imaged on an Eclipse 80i microscope (Nikon, TS2R-LS). The percentage of β-galactosidase-positive cells was calculated by counting the number of blue-stained cells in three random fields per sample ^4^.

*1.9. SPiDER-*β*Gal staining*

SPiDER-βGal staining was performed to detect senescence in BLM-treated A549 cells. A549 cells were seeded in 6-well plates and treated with 6-OHDA (100 μM), NAD^+^ (1 mM), PJ34 (5 μM), or their combination for 24 hours. After treatment, the cells were washed twice with PBS and fixed in 4% PFA for 10 minutes at room temperature. The cells were then washed with PBS and incubated with SPiDER-βGal working solution (DOJINDO, SG03) at 37°C for 30 minutes according to the manufacturer’s instructions. After incubation, the cells were washed again with PBS, and the nuclei were stained with Hoechst 33342 (10 μg/mL) for 5 minutes. The cells were then washed with PBS and imaged using a fluorescence microscope (Olympus IX73). The fluorescence intensity of SPiDER-βGal was quantified using ImageJ software by measuring the integrated density of fluorescence in three random fields per sample ^5^.

*1.10. JC-1 staining*

JC-1 staining was used to detect the MMP in 6-OHDA-treated SH-SY5Y cells. SH-SY5Y cells were seeded in 6-well plates and treated with 6-OHDA (1 mM), NAD^+^ (1 mM), PJ34 (5 μM), or their combination for 30 min. After treatment, the cells were washed twice with PBS and incubated with JC-1 staining solution (Beyotime, C2006) at 37°C for 20 min according to the manufacturer’s instructions. Following incubation, the cells were washed with JC-1 staining buffer and imaged immediately using a fluorescence microscope (Olympus IX73). JC-1 dye accumulates in mitochondria, forming aggregates (red fluorescence) in healthy cells with high mitochondrial membrane potential, while it remains in a monomeric form (green fluorescence) in cells with low membrane potential. The ratio of red to green fluorescence intensity was measured using ImageJ software to assess the MMP. Three random fields per sample were analyzed to ensure statistical relevance ^6^.

*1.11. MitoTracker staining*

MitoTracker Red CMXRos staining was used to evaluate mitochondrial morphology in 6-OHDA-induced SH-SY5Y cells. Briefly, SH-SY5Y cells were seeded onto glass-bottom confocal dishes and treated with 6-OHDA (1 mM), NAD⁺ (1 mM), PJ34 (5 μM), or their combination for 30 minutes. Following treatment, the cells were washed twice with PBS and incubated with MitoTracker Red CMXRos (Beyotime, C1049B) at a final concentration of 200 nM in serum-free medium at 37°C for 30 minutes. Cells were then washed with PBS to remove excess dye and fixed with 4% paraformaldehyde for 10 minutes at room temperature. After additional PBS washes, the cells were mounted using antifade mounting medium containing DAPI for nuclear staining. Mitochondrial morphology was visualized using a Zeiss LSM 980 confocal microscope (63× oil immersion objective). For quantitative analysis, confocal images were converted to binary format using ImageJ software (NIH, Bethesda, USA). The “Skeletonize” and “Analyze Skeleton” plugins were applied to extract mitochondrial structures. Average mitochondrial length per cell was calculated as the total branch length divided by the number of branches^7^.

*1.12. GFP-RFP-LC3 fluorescence analysis*

GFP-RFP-LC3 fluorescence analysis was used to assess autophagic flux in 6-OHDA-induced U87 cells ^8^. Stable RFP-GFP-LC3 U87 cells were seeded in 6-well plates and treated with 6-OHDA (1 mM), NAD^+^ (1 mM), PJ34 (5 μM), or their combination for 30 min. Following treatment, the cells were washed twice with PBS and fixed with 4% PFA for 10 min at room temperature. The fixed cells were then washed again with PBS and mounted with antifade mounting medium containing DAPI to stain the nuclei. Autophagosomes (yellow puncta) and autolysosomes (red puncta) were observed using a confocal microscope at 63× magnification. The GFP signal is sensitive to acidic conditions and is quenched in autolysosomes, while the RFP signal is stable, allowing differentiation between autophagosomes and autolysosomes. Images were acquired, and the number of GFP-RFP-LC3 puncta per cell was quantified using ImageJ software (NIH). Three random fields per sample were analyzed to evaluate the effects of different treatments on autophagic flux. The ratio of GFP-LC3 to RFP-LC3 puncta was used to assess the induction of autophagy and the subsequent maturation and degradation of autophagosomes.

*1.13. Lysosomal activity*

Lysosomal activity in 6-OHDA-induced SH-SY5Y cells was assessed using the Lysosomal Acidic pH Detection Kit-Green/Deep Red (DOJINDO, Japan). SH-SY5Y cells were seeded in 6-well plates and treated with 6-OHDA (1 mM), NAD^+^ (1 mM), PJ34 (5 μM), or their combination for 30 min. After treatment, the cells were washed twice with PBS and incubated with the Lysosomal Acidic pH Detection Kit reagent according to the manufacturer’s instructions. Briefly, the cells were incubated with the fluorescent dye for 30 minutes at 37°C to stain lysosomes with an acidic pH. Following incubation, the cells were washed with PBS to remove excess dye. The fluorescence intensity, indicating lysosomal activity, was measured using a fluorescence microscope. Images were captured, and the fluorescence intensity ratio of green to deep red was analyzed using ImageJ software. Three random fields per sample were evaluated to determine lysosomal activity.

*1.14. Plasmid transfection*

For plasmid transfection, BV-2 cells were seeded in 6-well plates at a density of 2 × 10^5^ cells per well and cultured until they reached 70-80% confluence. The cells were transfected with the following plasmids: pGFP-N1-NLRP3, pEGFP-N1-caspase-1, pmCherry-C1-ASC, and AcGFP1-N1-GSDMD using Lipomaster 2000 Transfection Reagent (Vazyme, TL201-01) according to the manufacturer’s instructions. Briefly, for each transfection, 2.5 µg of plasmid DNA and 5 µL of Lipomaster 2000 were diluted separately in 250 µL of Opti-MEM (Gibco, 31985070) without serum. The diluted DNA and Lipomaster were gently mixed and incubated for 15 minutes at room temperature to allow the formation of DNA-liposome complexes. The complexes were then added dropwise to the cells and mixed gently by rocking the plate back and forth. The cells were incubated at 37°C in a 5% CO_2_ incubator for 6 hours, after which the medium was replaced with fresh complete medium. The expression of the transfected genes was confirmed at 18-24 hours posttransfection using fluorescence microscopy.

*1.15. C. elegans strains and culture*

*The C. elegans* strains used in this study included N2 (wild-type), BZ555 (Pdat-1::GFP), NL5901 (Punc-54::α-synuclein::YFP), BC12921 (Pvha-6::GFP::LGG-1), SJ4103 [myo-3::GFP(mit)] and MAH215 (Pmyo-3::mCherry::GFP::LGG-1). The worms were maintained on nematode growth medium (NGM) plates seeded with *Escherichia coli* OP50 at 20°C under standard laboratory conditions. To synchronize the worm population, gravid adults were bleached using a solution containing sodium hypochlorite and sodium hydroxide, and the resulting eggs were allowed to hatch overnight in M9 buffer. Synchronized L1 larvae were then transferred to fresh NGM plates and grown to the L4 stage before being used in experiments.

*1.16. Measurement of body length and width in the C. elegans N2 strain*

To measure the body length and width of *the C. elegans* N2 strain, synchronized L4 larvae were treated with NAD^+^ (1 mM), PJ34 (40 μM), or their combination. The worms were incubated on the treated plates for 1, 3, or 5 days. At each time point, worms were picked and washed with M9 buffer to remove bacteria and debris. The cleaned worms were then transferred to a 2% agarose pad on a microscope slide and immobilized with 10 mM sodium azide. Images of the worms were captured using a fluorescence microscope (Leica Microsystems GmbH, Germany). The body length and width of each worm were measured using ImageJ software (NIH). At least 20 worms were measured per condition to ensure statistical relevance ^3^.

*1.17. Pharyngeal pumping assay in N2 worms*

To assess the effects of NAD^+^ (1 mM), PJ34 (40 μM), and their combination on worm mobility, the pharyngeal pumping rate in N2 worms was measured ^9^. Synchronized L4 N2 worms were transferred to NGM plates supplemented with the indicated treatments and incubated for the indicated times. Worms were then placed on a fresh NGM plate, and the rate of pharyngeal pumping, defined as the contraction of the terminal bulb, was counted under a stereo fluorescence microscopy (Leica, M205FA, Germany) for 20 seconds. This process was repeated for at least 20 worms per condition.

*1.18. Determination of lipofuscin content in N2 worms*

To determine the lipofuscin content in N2 worms, synchronized L4 larvae were transferred to NGM plates supplemented with NAD^+^ (1 mM), PJ34 (40 μM), or their combination and incubated for the indicated times. After treatment, the worms were washed three times with M9 buffer to remove any residual bacteria. Worms were then transferred to a 2% agarose pad on a microscope slide and immobilized with 10 mM sodium azide. Lipofuscin autofluorescence was observed using a fluorescence microscope (Leica Microsystems GmbH, Germany) with an excitation wavelength of 365 nm and an emission wavelength of 420 nm. Images were acquired, and the fluorescence intensity was quantified using ImageJ software (NIH). The relative lipofuscin content was calculated by measuring the mean fluorescence intensity in at least 20 worms per condition ^9^.

*1.19. Measurement of dopaminergic neuron viability in 6-OHDA-induced BZ555 worms*

To assess the viability of dopaminergic neurons in 6-OHDA-induced BZ555 worms, synchronized L4 larvae expressing GFP under the control of the dat-1 promoter (BZ555: Pdat-1::GFP) were treated with 6-OHDA (50 mM) in M9 buffer for 1 h in the dark with gentle shaking. After treatment, the worms were washed three times with M9 buffer to remove excess 6-OHDA and transferred to NGM plates supplemented with NAD^+^ (1 mM), PJ34 (40 μM), or their combination. L-DOPA (2 mM) was used as a positive control. After treatment, the worms were washed with M9 buffer to remove bacteria and debris. They were then transferred to a 2% agarose pad on a microscope slide and immobilized with 10 mM sodium azide. The viability of dopaminergic neurons was observed using a fluorescence microscope (Leica Microsystems GmbH, Germany) to detect GFP fluorescence. Images of the anterior region of the worms were captured, focusing on the head region where the dopaminergic neurons are located. The fluorescence intensity of GFP, indicating the viability of dopaminergic neurons, was quantified using ImageJ software (NIH) ^8^. At least 20 worms per condition were analyzed.

*1.20. Food-sensing ability assay in 6-OHDA-induced BZ555 worms*

6-OHDA-induced BZ555 worms were treated according to the above methods. After treatment, the food-sensing ability was assessed using a food race assay. A circular ring (2 cm in diameter) was drawn in the center of a fresh NGM plate, and the area inside the ring was seeded with a small amount of *E. coli* OP50 as the food source. Individual worms were placed at the edge of the plate, approximately 2 cm away from the food source, and the number of worms that reached the food source within 10 minutes was recorded. The assay was conducted under controlled environmental conditions (20°C) to ensure consistency, and at least 20 worms per condition were tested in triplicate to ensure statistical relevance. The results are presented as the percentage of worms that reached the food source within the given time ^10^.

*1.21. Measurement of α-synuclein in NL5901 worms*

To measure *α*-synuclein levels in NL5901 worms, synchronized L4 larvae expressing YFP-tagged α-synuclein under the control of the unc-54 promoter (NL5901: Punc-54::α-synuclein::YFP) were used. Worms were treated with NAD^+^ (1 mM), PJ34 (40 μM), or their combination, with L-DOPA (2 mM) used as a positive control. After treatment, the worms were washed three times with M9 buffer to remove residual bacteria. They were then transferred to a 2% agarose pad on a microscope slide and immobilized with 10 mM sodium azide. The expression of α-synuclein was observed using a fluorescence microscope (Leica Microsystems GmbH, Germany) with an excitation wavelength appropriate for YFP. Images were captured, and the GFP intensity, indicating the level of α-synuclein, was quantified using ImageJ software (NIH). At least 20 worms per condition were analyzed ^3^.

*1.22. Measurement of mobility in C. elegans*

N2, BZ555, and NL5901 worms were treated according to the above methods. After treatment, worm mobility was assessed by measuring body bends. For body bends, worms were placed individually on a 2% agarose pad on a microscope slide, and the number of body bends was counted under a stereo fluorescence microscope (Leica, M205FA, Germany) for 20 seconds, with at least 20 worms per condition measured ^3^.

*1.23. Measurement of mitochondrial integrity in 6-OHDA-induced SJ4103 worms*

To measure mitochondrial integrity in 6-OHDA-induced SJ4103 worms, synchronized L4 larvae expressing GFP-tagged mitochondrial proteins (SJ4103: Pmyo-3::GFP) were used. The worms were treated with 6-OHDA (50 mM) in M9 buffer for 1 hour in the dark with gentle shaking. After treatment, the worms were washed three times with M9 buffer to remove excess 6-OHDA and then transferred to NGM plates supplemented with NAD^+^ (1 mM), PJ34 (40 μM), or their combination. L-DOPA (2 mM) was used as a positive control. Following treatment, the worms were washed with M9 buffer to remove bacteria and debris. The worms were then transferred to a 2% agarose pad on a microscope slide and immobilized with 10 mM sodium azide. Mitochondrial integrity was observed using a fluorescence microscope (Leica Microsystems GmbH, Germany). Images were captured, focusing on the body wall muscle where mitochondrial networks are prominently visible ^11^.

*1.24. Measurement of p62 expression in 6-OHDA-induced BC12921 worms*

To measure p62 expression in 6-OHDA-induced BC12921 worms, synchronized L4 larvae expressing GFP-tagged p62 under the control of the vha-6 promoter (BC12921: Pvha-6::GFP::LGG-1) were used. Worms were treated with 6-OHDA (50 mM) in M9 buffer for 1 hour in the dark with gentle shaking. After treatment, worms were washed three times with M9 buffer to remove excess 6-OHDA and then transferred to NGM plates supplemented with NAD^+^ (1 mM), PJ34 (40 μM), or their combination. Following the specified treatment period, worms were washed with M9 buffer to remove bacteria and debris. They were then transferred to a 2% agarose pad on a microscope slide and immobilized with 10 mM sodium azide. The expression of p62 was observed using a fluorescence microscope (Leica Microsystems GmbH, Germany). Images were captured, and the GFP intensity, indicating the level of p62 expression, was quantified using ImageJ software (NIH). At least 20 worms per condition were analyzed ^8^.

*1.25. Measurement of autophagy flux in 6-OHDA-induced MAH215 worms*

To measure autophagy flux in 6-OHDA-induced MAH215 worms, synchronized L4 larvae expressing GFP- and RFP-tagged LGG-1 under the control of the myo-3 promoter (MAH215: Pmyo-3::mCherry::GFP::LGG-1) were used. Worms were treated with 6-OHDA (50 mM) in M9 buffer for 1 h in the dark with gentle shaking. After treatment, the worms were washed three times with M9 buffer to remove excess 6-OHDA and then transferred to NGM plates supplemented with NAD^+^ (1 mM), PJ34 (40 μM), or their combination. Following treatment, the worms were washed with M9 buffer to remove bacteria and debris. They were then transferred to a 2% agarose pad on a microscope slide and immobilized with 10 mM sodium azide. Autophagy flux was observed using a fluorescence microscope (Leica Microsystems GmbH, Germany) with appropriate excitation and emission wavelengths for GFP and RFP. The GFP signal is sensitive to acidic conditions and is quenched in autolysosomes, while the RFP signal is stable, allowing differentiation between autophagosomes (GFP-RFP puncta) and autolysosomes (RFP puncta). The number of GFP and RFP puncta was quantified using ImageJ software (NIH). At least 20 worms per condition were analyzed. The ratio of GFP to RFP puncta was calculated to assess autophagic flux ^12^.

*1.26. 6-OHDA-induced mice and drug administration*

To establish a mouse model of Parkinson’s disease (PD), male C57BL/6J mice (8–10 weeks old, 25–30 g) were housed under standard laboratory conditions (23 ± 2 °C, 12-hour light/dark cycle) with ad libitum access to food and water. All animal procedures were approved by the Institutional Animal Care and Use Committee of Southwest Medical University (approval number: 20240515-006). Under deep anesthesia with pentobarbital sodium (50 mg/kg, i.p.), mice were fixed in a stereotaxic apparatus (Stoelting Co., USA). A small cranial burr hole was drilled at the following coordinates relative to bregma: anteroposterior (AP) –2.9 mm, mediolateral (ML) +1.1 mm, and dorsoventral (DV) –4.5 mm. A 1 μL volume of 6-hydroxydopamine (6-OHDA, 3 μg/μL in 0.02% ascorbic acid-saline) was injected unilaterally into the right striatum at a rate of 0.2 μL/min using a 30-gauge Hamilton microsyringe. The needle was left in place for an additional 5 minutes to minimize reflux before being slowly withdrawn. Mice were monitored during recovery on a heating pad and provided softened food and 5% glucose-saline to maintain hydration and nutrition. Fourteen days post-surgery, lesion efficacy was verified by immunohistochemical analysis of tyrosine hydroxylase (TH) expression in the substantia nigra pars compacta (SNpc) and striatum. A successful lesion was defined as a ≥70% reduction in TH immunoreactivity in the lesioned hemisphere compared to the contralateral side, consistent with previously established thresholds. Apomorphine-induced rotational behavior (0.5 mg/kg, subcutaneous) was evaluated on day 14 post-lesion to assess motor asymmetry. Mice displaying more than 7 contralateral rotations per minute were considered to have developed a robust unilateral lesion and were included in the experimental analysis. To further validate the model, we confirmed that behavioral deficits correlated with dopaminergic neurodegeneration, as assessed by reduced TH immunoreactivity and decreased dopamine levels in the striatum (see updated Results section for details). Three weeks after 6-OHDA injection, mice were randomly divided into six treatment groups (n = 8 per group): control group (saline injection), 6-OHDA group (saline injection), 6-OHDA + NAD^+^ group (10 mg/kg, intraperitoneally, daily), 6-OHDA + PJ34 group (5 mg/kg, intraperitoneally, daily), 6-OHDA + NAD^+^ + PJ34 group (10 mg/kg NAD^+^ + 5 mg/kg PJ34, intraperitoneally, daily), and 6-OHDA + L-DOPA group (10 mg/kg, intraperitoneally, daily). Treatments were administered once daily for four consecutive weeks ^8^.

*1.27. Apomorphine-induced rotation test*

An apomorphine-induced rotation test was conducted to assess the severity of unilateral 6-OHDA lesions in PD model mice. Male C57BL/6 mice that had undergone 6-OHDA lesion surgery and subsequent treatments were used for this test. One week before the completion of the treatment regimen, the mice were acclimatized to the testing environment for 30 minutes. The apomorphine hydrochloride (0.5 mg/kg, subcutaneously) was freshly prepared and administered to each mouse. Following apomorphine injection, the mice were placed in a cylindrical testing chamber (diameter: 20 cm) to allow free movement. The number of full body turns (360°) made by each mouse in the direction contralateral to the lesioned side (ipsilateral turns were considered nonspecific and not counted) was recorded over a 30-minute period. A full turn was defined as the mouse completing a 360° rotation. The total number of contralateral rotations was recorded for each mouse ^8^.

*1.28. Force swimming test*

The forced swimming test was conducted to evaluate the effects of NAD^+^, PJ34, and their combination on depressive-like behavior in 6-OHDA-induced mice. Male C57BL/6 mice that had undergone 6-OHDA lesion surgery and subsequent treatments were used for this test. One week before the completion of the treatment regimen, the mice were acclimatized to the testing environment. Each mouse was placed individually in a transparent glass cylinder (height: 25 cm, diameter: 20 cm) filled with water (25±2°C) to a depth of 15 cm, ensuring that the mouse could not touch the bottom or escape. The mice were observed for a total of 2 minutes, with the initial 1 minute considered the acclimatization period and the last 1 minute recorded for analysis. The duration of immobility, defined as the time spent floating without struggling and only making movements necessary to keep the head above water, was recorded during the last minute ^8^.

*1.29. Forelimb hanging test*

The forelimb hanging test was conducted to evaluate the effects of NAD^+^, PJ34, and their combination on muscle strength and motor coordination in 6-OHDA-induced mice. Male C57BL/6 mice that had undergone 6-OHDA lesion surgery and subsequent treatments were used for this test. One week before the completion of the treatment regimen, the mice were acclimatized to the testing environment. Each mouse was gently placed on a metal wire grid (2 mm diameter) suspended 70 cm above a soft bedding surface, allowing the mouse to grasp the wire grid with its forelimbs. The time that each mouse remained in suspension was recorded, with a maximum cutoff time of 1 minute. If a mouse fell before the cutoff time, the trial was repeated up to three times, and the longest hanging time was used for analysis ^8^.

*1.30. Immunohistochemistry*

Immunohistochemistry was performed to evaluate the expression of specific proteins in the brain tissues of 6-OHDA-induced mice. After treatment, the mice were deeply anesthetized with pentobarbital (50 mg/kg, intraperitoneally) and perfused transcardially with cold saline followed by 4% PFA in 0.1 M phosphate buffer (pH 7.4). The brains were carefully removed, postfixed in 4% paraformaldehyde at 4°C overnight, cryoprotected in 30% sucrose solution in PBS until they sank, embedded in optimal cutting temperature (OCT) compound, and sectioned coronally at 30 μm thickness using a cryostat. The brain sections were rinsed in PBS, incubated in 0.3% hydrogen peroxide for 30 minutes to block endogenous peroxidase activity, permeabilized with 0.3% Triton X-100 in PBS for 15 minutes, and blocked with 5% normal goat serum for 1 hour at room temperature. The sections were incubated overnight at 4°C with primary antibodies against TH, GFAP, and Iba1. After washing, the sections were incubated with biotinylated secondary antibodies for 1 hour at room temperature, followed by incubation with avidin-biotin complex (ABC) solution for 1 hour. Immunoreactivity was visualized using diaminobenzidine (DAB) as the chromogen. The sections were counterstained with hematoxylin, dehydrated through a graded series of ethanol, cleared in xylene, and coverslipped with mounting medium. Images of stained sections were captured using a digital slide scanner (KFBIO, KF-PRO-002), and the expression levels of specific proteins were quantified using ImageJ software (NIH). At least three sections per brain and three brains per group were analyzed.

*1.31. Statistical analysis*

All statistical analyses were performed using GraphPad Prism 9.0 (GraphPad Software, USA). Data are presented as mean ± standard deviation (SD). Prior to hypothesis testing, data distribution normality was assessed using the Shapiro–Wilk test, and homogeneity of variance was evaluated using Levene’s test. For comparisons among multiple groups, one-way ANOVA was applied, followed by Tukey’s post hoc test for pairwise comparisons. In cases where data did not meet parametric assumptions, nonparametric Kruskal–Wallis tests were conducted with Dunn’s post hoc test. Effect sizes (η² or Cohen’s d) were calculated and reported where applicable to complement p-values and reflect the magnitude of observed effects. Statistical significance was set at p < 0.05, with notation as follows: p < 0.05 (*), p < 0.01 (**), and p < 0.001 (***). All image quantifications (e.g., fluorescence intensity, band densitometry, mitochondrial length) were performed using ImageJ (NIH) with blinded ROI selection. Each experimental condition was replicated at least three times independently unless otherwise specified.

**References**

1. Teng JF, Mei QB, Zhou XG, et al. Polyphyllin VI Induces Caspase-1-Mediated Pyroptosis via the Induction of ROS/NF-κB/NLRP3/GSDMD Signal Axis in Non-Small Cell Lung Cancer. *Cancers (Basel).* 2020;12(1).

2. Qiu WQ, Pan R, Tang Y, et al. Lychee seed polyphenol inhibits Aβ-induced activation of NLRP3 inflammasome via the LRP1/AMPK mediated autophagy induction. *Biomed Pharmacother.* 2020;130:110575.

3. Zhu FD, Wang BD, Qin DL, et al. Carpesii fructus extract exhibits neuroprotective effects in cellular and Caenorhabditis elegans models of Parkinson's disease. *CNS Neurosci Ther.* 2024;30(4):e14515.

4. Neri F, Basisty N, Desprez PY, Campisi J, Schilling B. Quantitative Proteomic Analysis of the Senescence-Associated Secretory Phenotype by Data-Independent Acquisition. *Curr Protoc.* 2021;1(2):e32.

5. Tanino R, Tsubata Y, Harashima N, Harada M, Isobe T. Novel drug-resistance mechanisms of pemetrexed-treated non-small cell lung cancer. *Oncotarget.* 2018;9(24):16807-16821.

6. Sun X, Wu A, Kwan Law BY, et al. The active components derived from Penthorum chinensePursh protect against oxidative-stress-induced vascular injury via autophagy induction. *Free Radic Biol Med.* 2020;146:160-180.

7. Valente AJ, Maddalena LA, Robb EL, Moradi F, Stuart JA. A simple ImageJ macro tool for analyzing mitochondrial network morphology in mammalian cell culture. *Acta Histochem.* 2017;119(3):315-326.

8. Wu AG, Pan R, Law BY, et al. Targeting autophagy as a therapeutic strategy for identification of liganans from Peristrophe japonica in Parkinson's disease. *Signal Transduct Target Ther.* 2021;6(1):67.

9. Long T, Tang Y, He YN, et al. Citri Reticulatae Semen Extract Promotes Healthy Aging and Neuroprotection via Autophagy Induction in Caenorhabditis elegans. *J Gerontol A Biol Sci Med Sci.* 2022;77(11):2186-2194.

10. He CL, Tang Y, Chen X, et al. Folium Hibisci Mutabilis extract, a potent autophagy enhancer, exhibits neuroprotective properties in multiple models of neurodegenerative diseases. *Phytomedicine.* 2023;109:154548.

11. Feng X, Wang X, Zhou L, Pang S, Tang H. The impact of glucose on mitochondria and life span is determined by the integrity of proline catabolism in Caenorhabditis elegans. *J Biol Chem.* 2023;299(2):102881.

12. Jung R, Lechler MC, Fernandez-Villegas A, et al. A safety mechanism enables tissue-specific resistance to protein aggregation during aging in C. elegans. *PLoS Biol.* 2023;21(9):e3002284.

**
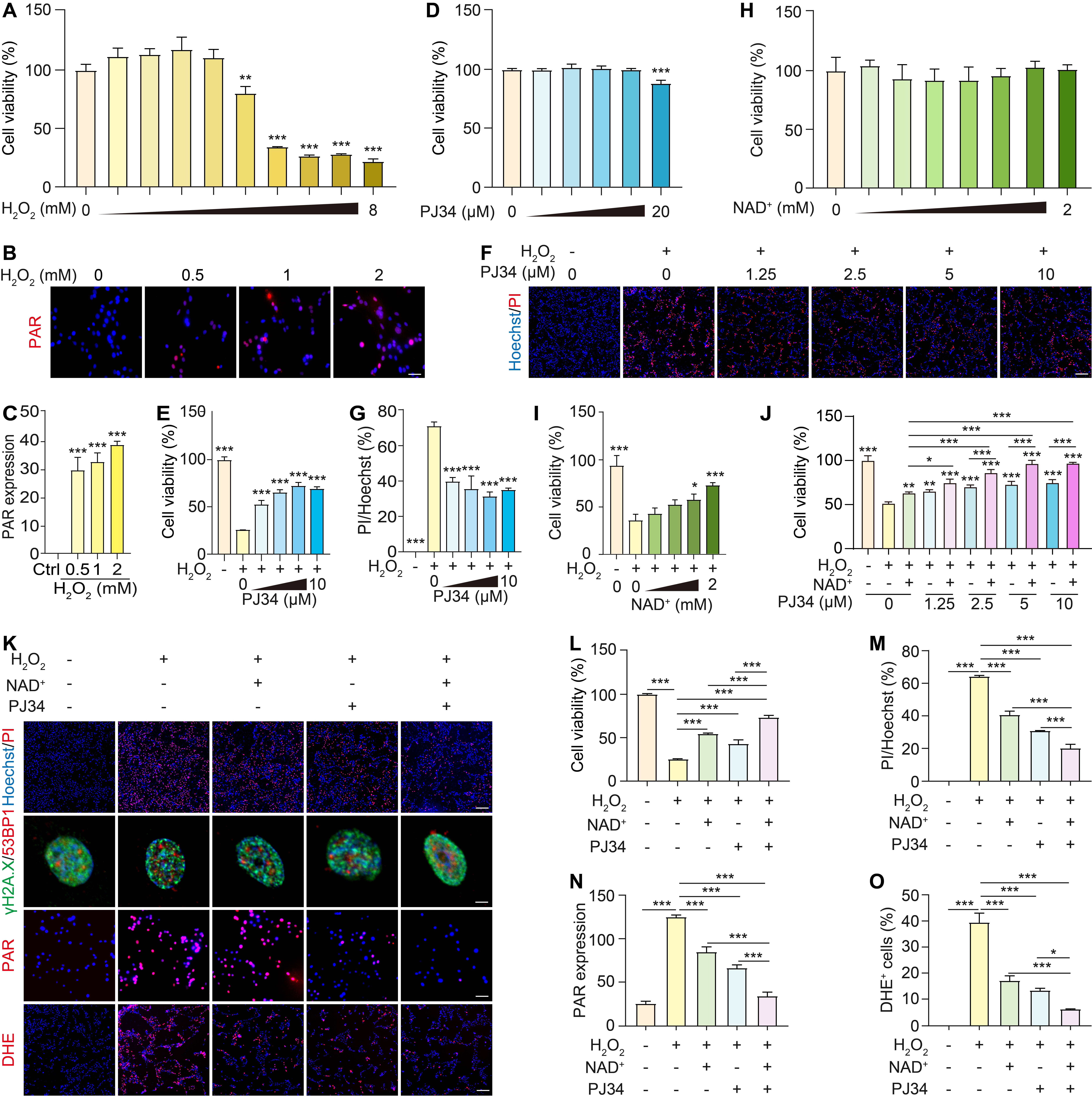
**

**Fig. S1. Effects of NAD^+^, PJ34, and their combination on H_2_O_2_-induced cytotoxicity in SH-SY5Y cells.** (A) Cell viability after exposure to increasing concentrations of H_2_O_2_ (n = 3). (B) Immunofluorescence images showing PAR expression (red) in neuronal cells treated with increasing concentrations of H_2_O_2_. Nuclei are stained with Hoechst (blue). Magnification: 40×, Scale bar = 50 μm. (C) Quantification of PAR expression in SH-SY5Y cells exposed to H_2_O_2_ (n = 3). (D) Cell viability after exposure to increasing concentrations of PJ34 (n = 3). (E) Viability of SH-SY5Y cells treated with H_2_O_2_ (1 mM) and increasing concentrations of PJ34 (1.25, 2.5, 5, and 10 μM) (n = 3). (F) Fluorescence images showing PI (red) and Hoechst (blue) staining in SH-SY5Y cells treated with H_2_O_2_ (1 mM) and PJ34 (1.25, 2.5, 5, and 10 μM). Magnification: 10×, Scale bar = 200 μm. (G) Quantification of the PI/Hoechst ratio in SH-SY5Y cells treated with H_2_O_2_ and PJ34 (n = 3). (H) Cell viability after exposure to increasing concentrations of NAD^+^ (n = 3). (I) Cell viability assay of SH-SY5Y cells treated with H_2_O_2_ (1 mM) and increasing concentrations of NAD^+^ (0.25, 0.5, 1, and 2 mM) (n = 3). (J) Cell viability assay of SH-SY5Y cells treated with H_2_O_2_ (1 mM), NAD^+^ (1 mM), PJ34 (1.25, 2.5, 5, and 10 μM) or their combination (n = 3). (K) Fluorescence images showing Hoechst/PI, γH2A.X/53BP1, PAR, and DHE staining in SH-SY5Y cells treated with H_2_O_2_, NAD^+^, PJ34 or their combination. Magnification: 10×, Scale bar = 200 μm, 40×, Scale bar = 50 μm, 63×, Scale bar = 32 μm. (L) Cell viability assay of SH-SY5Y cells treated with H_2_O_2_, NAD^+^ (1 mM), PJ34 (5 μM), or their combination (n = 3). (M-O) Quantification of the PI/Hoechst ratio, PAR expression, and DHE-positive cells (n = 3). Data were analyzed by a one-way ANOVA followed by Dunnett's multiple comparisons test was used. *** *p* < 0.05, **** *p* < 0.01, and ***** *p* < 0.001.

**
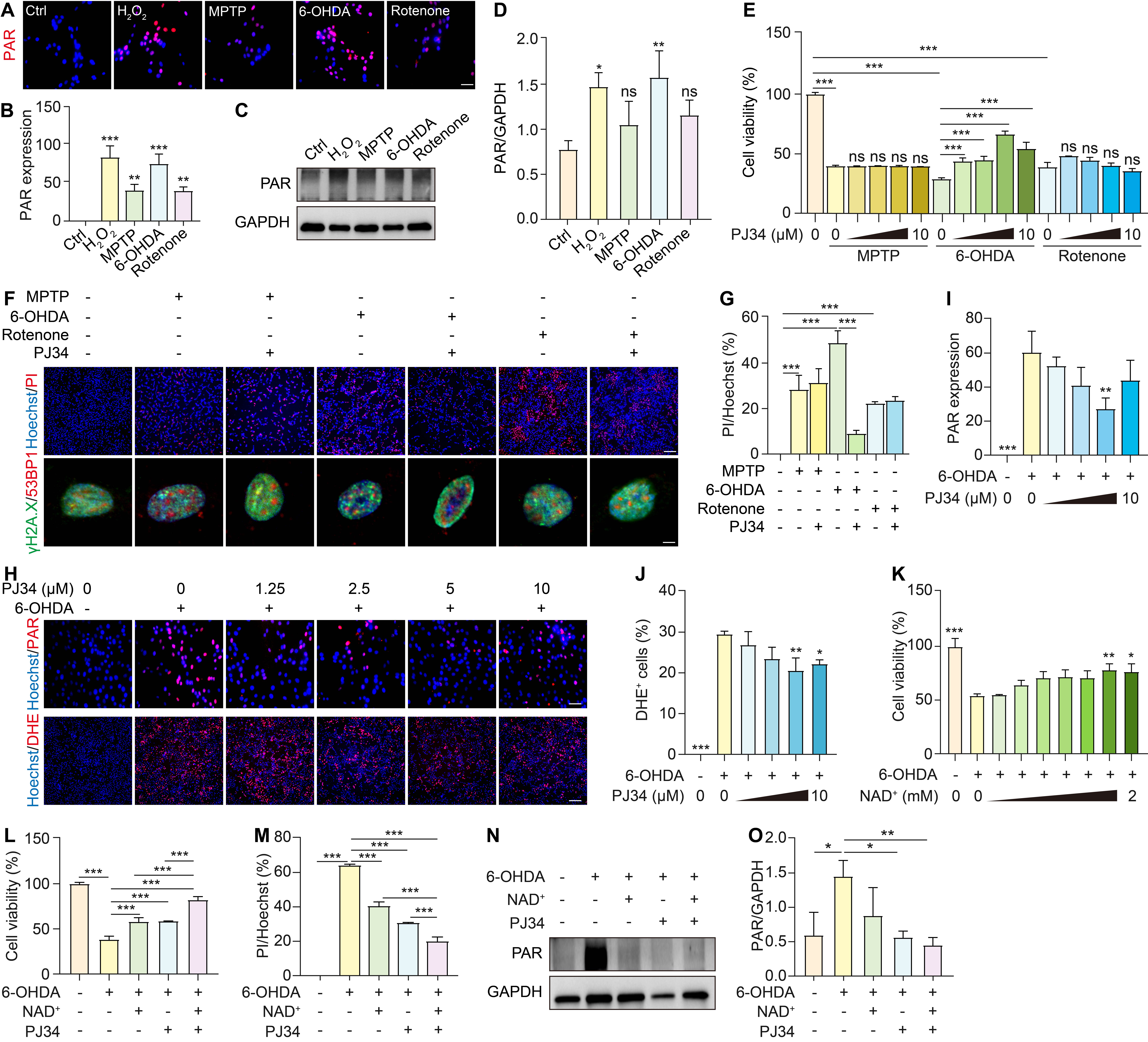
Fig. S2. Effects of NAD^+^, PJ34, and their combination on neurotoxicity induced by MPTP, 6-OHDA, and rotenone in SH-SY5Y cells.** (A) Immunofluorescence images showing PAR expression (red) in SH-SY5Y cells treated with MPTP (1 mM), 6-OHDA (1 mM), and rotenone (1 mM). Nuclei are stained with Hoechst (blue). Magnification: 40×, Scale bar = 50 μm. (B) Quantification of PAR expression in SH-SY5Y cells (n = 3). (C) Western blot analysis of PAR in SH-SY5Y cells treated with MPTP, 6-OHDA, and rotenone. Original and non-processed Western blot images were provided in Fig. S10A. (D) Densitometric analysis of the PAR/GAPDH ratio (n = 3). (E) Viability of SH-SY5Y cells treated with MPTP, 6-OHDA, and rotenone and increasing concentrations of PJ34 (0, 1.25, 2.5, 5, or 10 μM) (n = 3). (F) Fluorescence images showing Hoechst/PI and γH2A.X/53BP1 staining in SH-SY5Y cells treated with MPTP, 6-OHDA, rotenone, and PJ34 (5 μM). Magnification: 10×, Scale bar = 200 μm, 63×, Scale bar = 32 μm. (G) Quantification of the PI/Hoechst ratio in cells (n = 3). (H) Immunofluorescence images showing PAR expression and DHE intensity in SH-SY5Y cells treated with 6-OHDA (1 mM) and increasing concentrations of PJ34 (0, 1.25, 2.5, 5, or 10 μM). Magnification: 40× and 10×, Scale bar = 50 μm and 200 μm. (I) Quantification of PAR expression (n = 3). (J) Quantification of the percentage of DHE-positive cells (n = 3). (K) Viability of SH-SY5Y cells treated with 6-OHDA in the presence or absence of NAD^+^ (0-2 mM) (n = 3). (L) Viability of SH-SY5Y cells treated with 6-OHDA (1 mM), NAD^+^ (1 mM), PJ34 (5 μM), or their combination (n = 3). (M) Quantification of the PI/Hoechst ratio in SH-SY5Y cells treated with 6-OHDA (1 mM), NAD^+^ (1 mM), PJ34 (5 μM), or their combination (n = 3). (N) Western blot analysis of PAR in SH-SY5Y cells treated with 6-OHDA, NAD^+^, PJ34, or their combination. Original and non-processed Western blot images were provided in Fig. S10B. (O) Densitometric analysis of the PAR/GAPDH ratio (n = 3). Data were analyzed by a one-way ANOVA followed by Dunnett's multiple comparisons test was used. ns = not significant; *** *p* < 0.05, **** *p* < 0.01, and ***** *p* < 0.001.

**
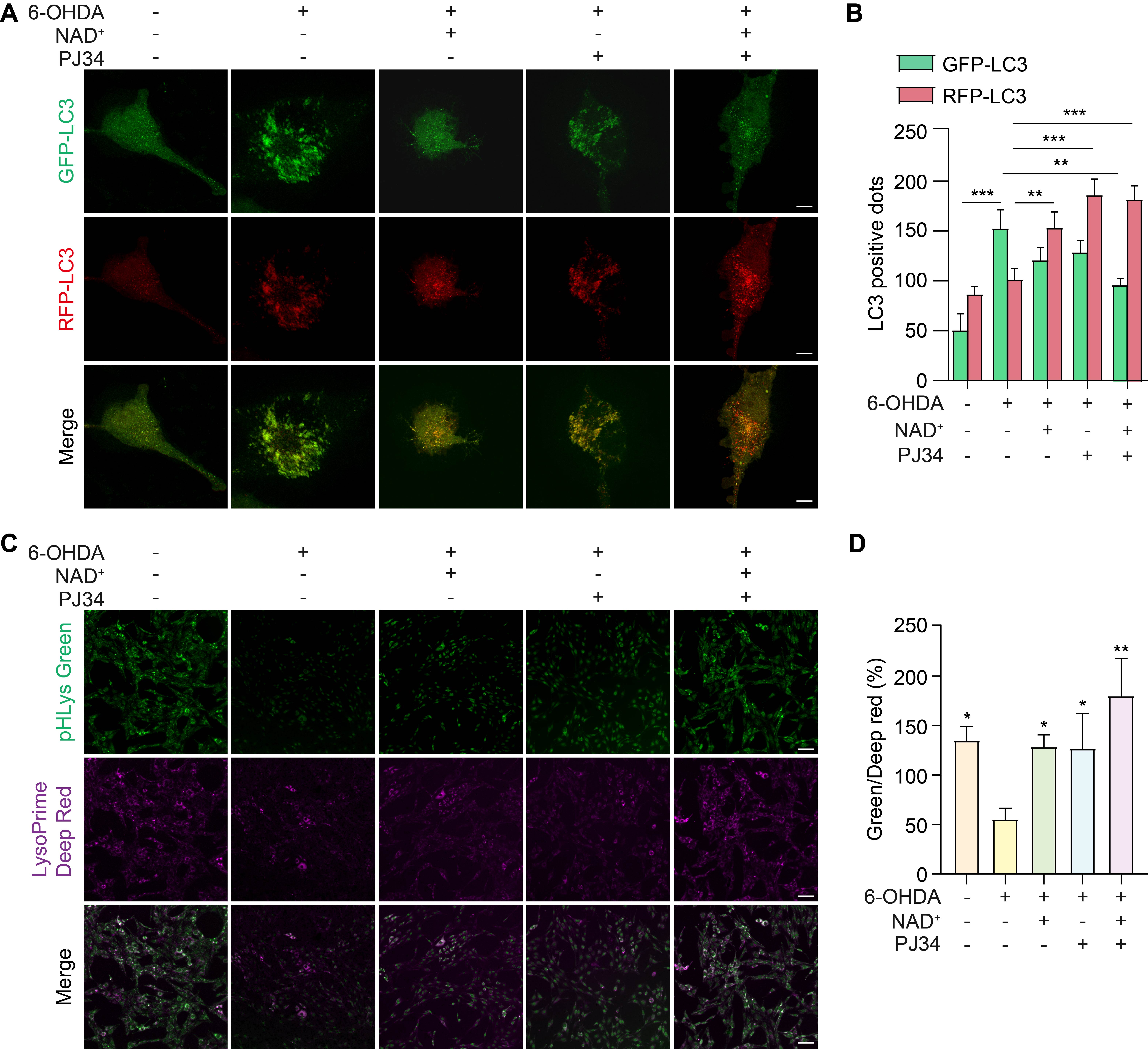
**

**Fig. S3. Effect of PJ34, NAD^+^, and their combination on autophagy-lysosme pathway in 6-OHDA-induced stable RFP-GFP-LC3 U87 cells and SH-SY5Y cells.** (A) Representative fluorescence images showing GFP-LC3 and RFP-LC3 puncta in 6-OHDA-induced stable RFP-GFP-LC3 U87 cells treated with NAD^+^, PJ34, or their combination. Magnification: 63×, Scale bar: 32 μm. (B) Quantification of GFP-LC3 and RFP-LC3 puncta (n = 3). (C) Representative images of SH-SY5Y cells showing pH-Lys Green and LysoPrime Deep Red staining along with their colocalization. Magnification: 20×, Scale bar: 100 μm. (D) Quantification of the green/deep red fluorescence ratio (n = 3). Data were analyzed by a one-way ANOVA followed by Dunnett's multiple comparisons test was used. *** *p* < 0.05, **** *p* < 0.01, and ***** *p* < 0.001.


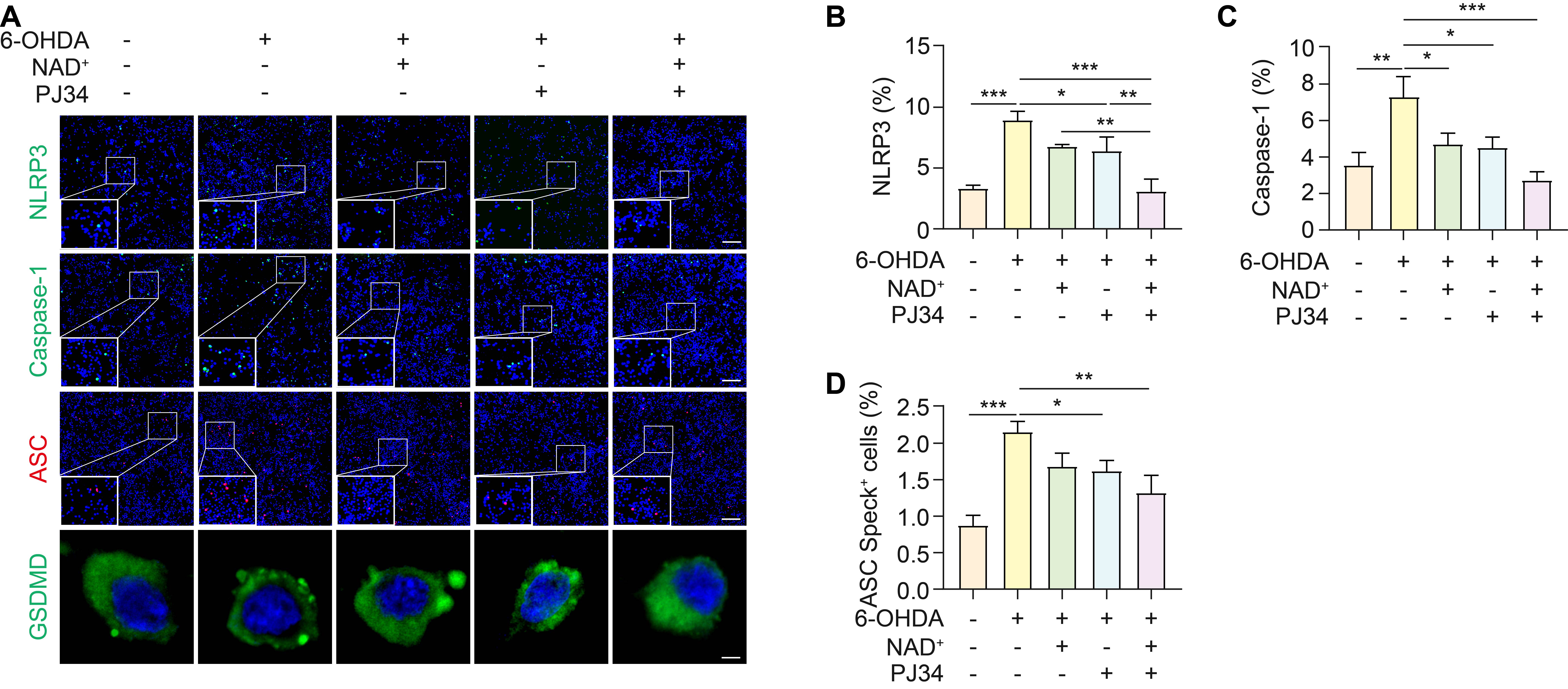


**Fig. S4** Effects of NAD^+^, PJ34, and their combination on NLRP3 inflammasome activation in 6-OHDA-induced BV-2 cells. (A) Representative images showing the fluorescence of NLRP3, Caspase-1, ASC, and GSDMD in 6-OHDA-induced BV-2 cells treated with NAD^+^, PJ34, or their combination. Magnification: 10× and 63×, Scale bar: 200 μm and 32 μm. (B-D) Quantification of NLRP3, Caspase-1, and ASC speck-positive cells (n = 3). Data were analyzed by a one-way ANOVA followed by Dunnett's multiple comparisons test was used. ns = not significant; *** *p* < 0.05, **** *p* < 0.01, and ***** *p* < 0.001.


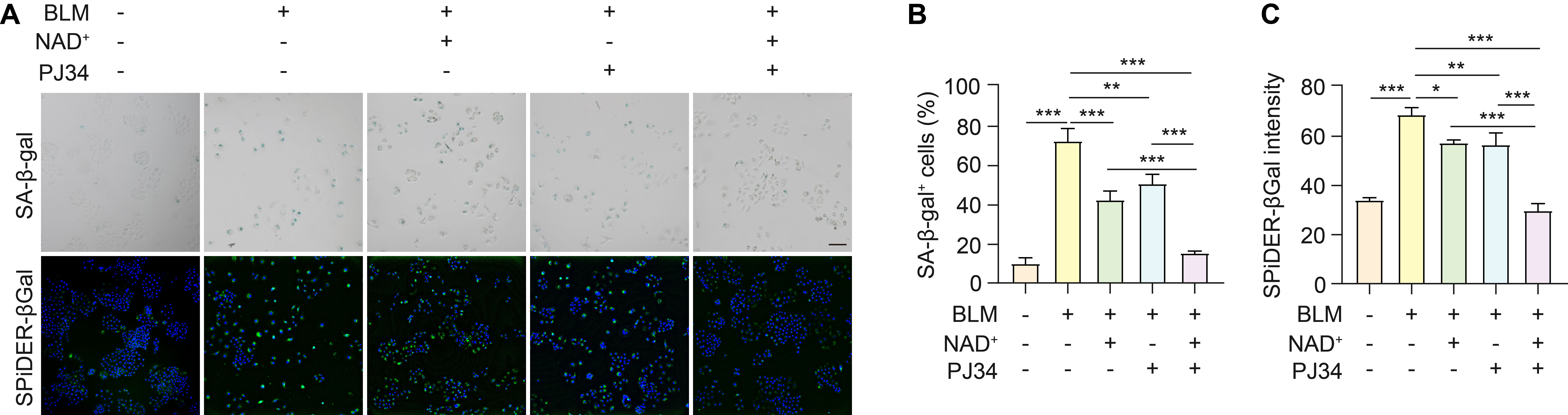


**Fig. S5** Effects of NAD^+^, PJ34, and their combination on cellular senescence in BLM-induced A549 cells. (A) Representative images of SA-β-gal and SPiDER-βGal staining in BLM-induced A549 cells treated with NAD^+^, PJ34, or their combination. Magnification: 20× and 10×, Scale bar = 100 μm and 200 μm. (B, C) Quantification of SA-β-gal-positive cells (%) and the SPiDER-βGal fluorescence intensity (n = 3). Data were analyzed by a one-way ANOVA followed by Dunnett's multiple comparisons test was used. ns = not significant; *** *p* < 0.05, **** *p* < 0.01, and ***** *p* < 0.001.


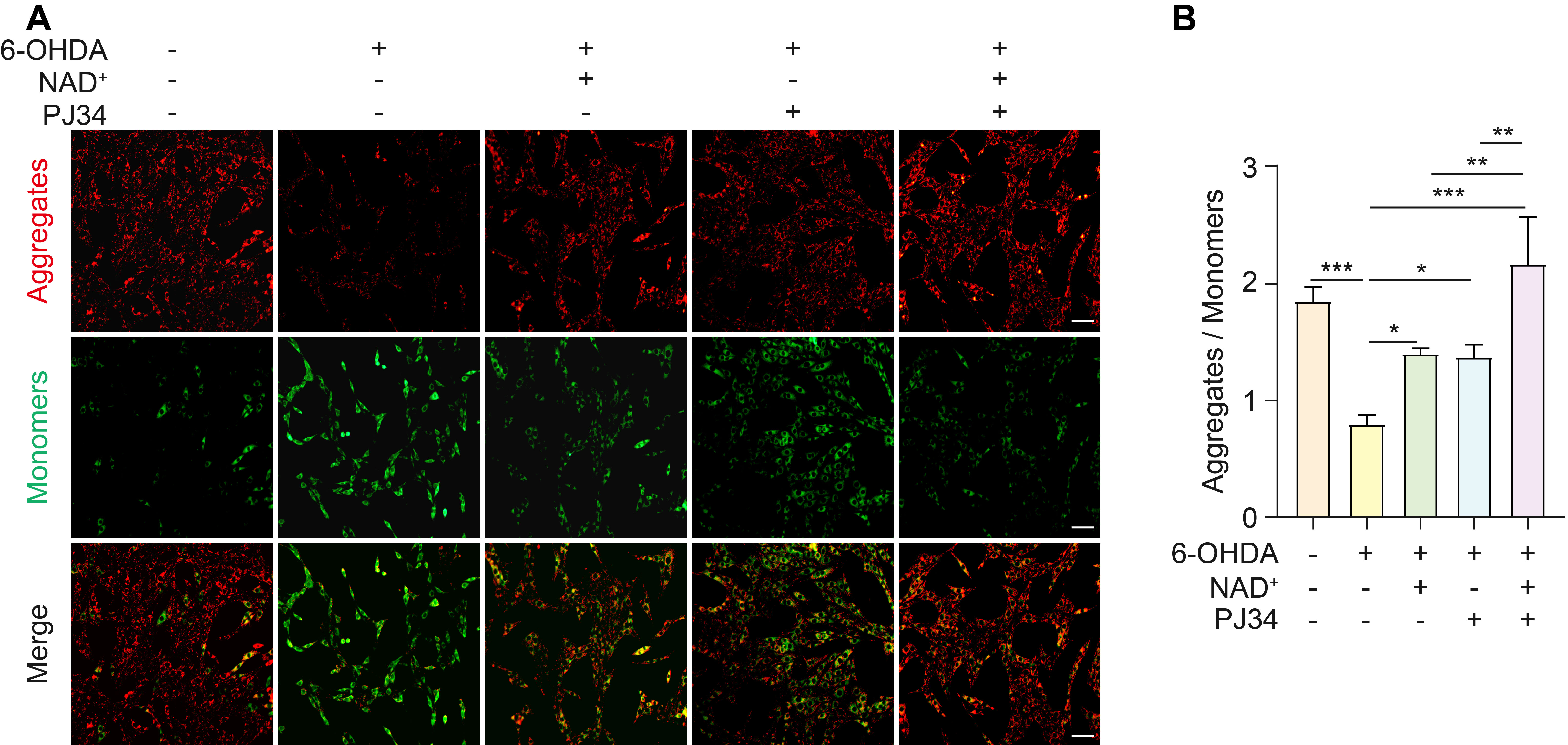


**Fig. S6** Effects of NAD^+^, PJ34, and their combination on mitochondrial membrane potential in 6-OHDA-induced SH-SY5Y cells. (A) Representative JC-1-stained images in 6-OHDA-induced SH-SY5Y cells treated with NAD^+^, PJ34, or their combination. JC-1 aggregates are labeled in red, JC-1 monomers in green, and merged images show overlap. Magnification: 20×, Scale bar: 100 μm. (B) Quantification of the JC-1 aggregate/monomer ratio (n = 3). Data were analyzed by a one-way ANOVA followed by Dunnett's multiple comparisons test was used. ns = not significant; *** *p* < 0.05, **** *p* < 0.01, and ***** *p* < 0.001.

**

Fig. S7.** Neuroprotective effects of NAD^+^, PJ34, and their combination in 6-OHDA-induced *C. elegans*. (A) Representative images showing the body length and width of N2 strains treated with NAD^+^, PJ34, or their combination on days 1, 3, and 5. Magnification: 10×, Scale bar = 200 μm. (B, C) Quantification of the body length and body width of N2 worms (n = 20). (D, E) Quantification of the number of body bends per 20 seconds and the pumping rate per 20 seconds of N2 worms under different treatments (n = 20). (F) Representative images showing the lipofuscin content in N2 worms subjected to different treatments. Magnification: 10×, Scale bar = 200 μm. (G) Quantification of the lipofuscin content (a.u.) in N2 worms (n = 20). (H) Quantification of the slowing rate in 6-OHDA-induced BZ555 worms treated with NAD^+^, PJ34, or their combination (n = 20). (I) Quantification of the number of body bends per 20 seconds in 6-OHDA-induced BZ555 worms treated with NAD^+^, PJ34, or their combination (n = 20). (J) Representative images showing GFP::p62 intensity in 6-OHDA-induced BC12921 worms treated with NAD^+^, PJ34, or their combination. Magnification: 20×, Scale bar = 100 μm. (K) Quantification of GFP intensity (n = 20). (L) Quantification of the number of body bends per 20 seconds in NL5901 *C. elegans* treated with NAD^+^, PJ34, or their combination (n = 20). (M) Representative fluorescence images show the GFP::α-synuclein expression in NL5901 *C. elegans* treated with NAD^+^, PJ34, their combination, and L-Dopa (2 mM). Magnification: 10×, Scale bar: 200 μm. (N) Quantitative analysis of GFP intensity in NL5901 *C. elegans* (n = 20). Data were analyzed by a one-way ANOVA followed by Dunnett's multiple comparisons test was used. ns = not significant; *** *p* < 0.05, **** *p* < 0.01, and ***** *p* < 0.001.





**Figure 8. Neuroprotective effects of NAD^+^, PJ34, and their combination in 6-OHDA-induced mice.** (A) Schematic representation of 6-OHDA injection in the brain and the experimental timeline of this study. (B-D) Behavioral performance evaluation, including rotation behavior, swimming time, and suspension time in mice (n = 6). (E) Western blot analysis of PAR and its quantification in the brain tissue of different treatment groups (n = 3). Original and non-processed Western blot images were provided in Fig. S10. (F) Immunofluorescence images showing expression of LC3, Lamp2, and COX-2 in the cortex region, with zoomed-in sections highlighting differences among treatment groups. Magnification: 40×, Scale bar = 50 μm. (G-I) Quantification of the immunofluorescence intensity of LC3 (G), Lamp2 (H), and COX-2 (I) (n = 3). (J, K) Immunohistochemical staining for GFAP and Iba1 showing astrocyte and microglial activation in the different groups. Magnification: 20×, Scale bar = 100 μm. (L, M) Quantification of GFAP^+^ cells and Iba1^+^ cells indicating the percentage of activated astrocytes and microglia in each group (n = 3). Data were analyzed by a one-way ANOVA followed by Dunnett's multiple comparisons test was used. ns = not significant; *** *p* < 0.05, **** *p* < 0.01, and ***** *p* < 0.001.


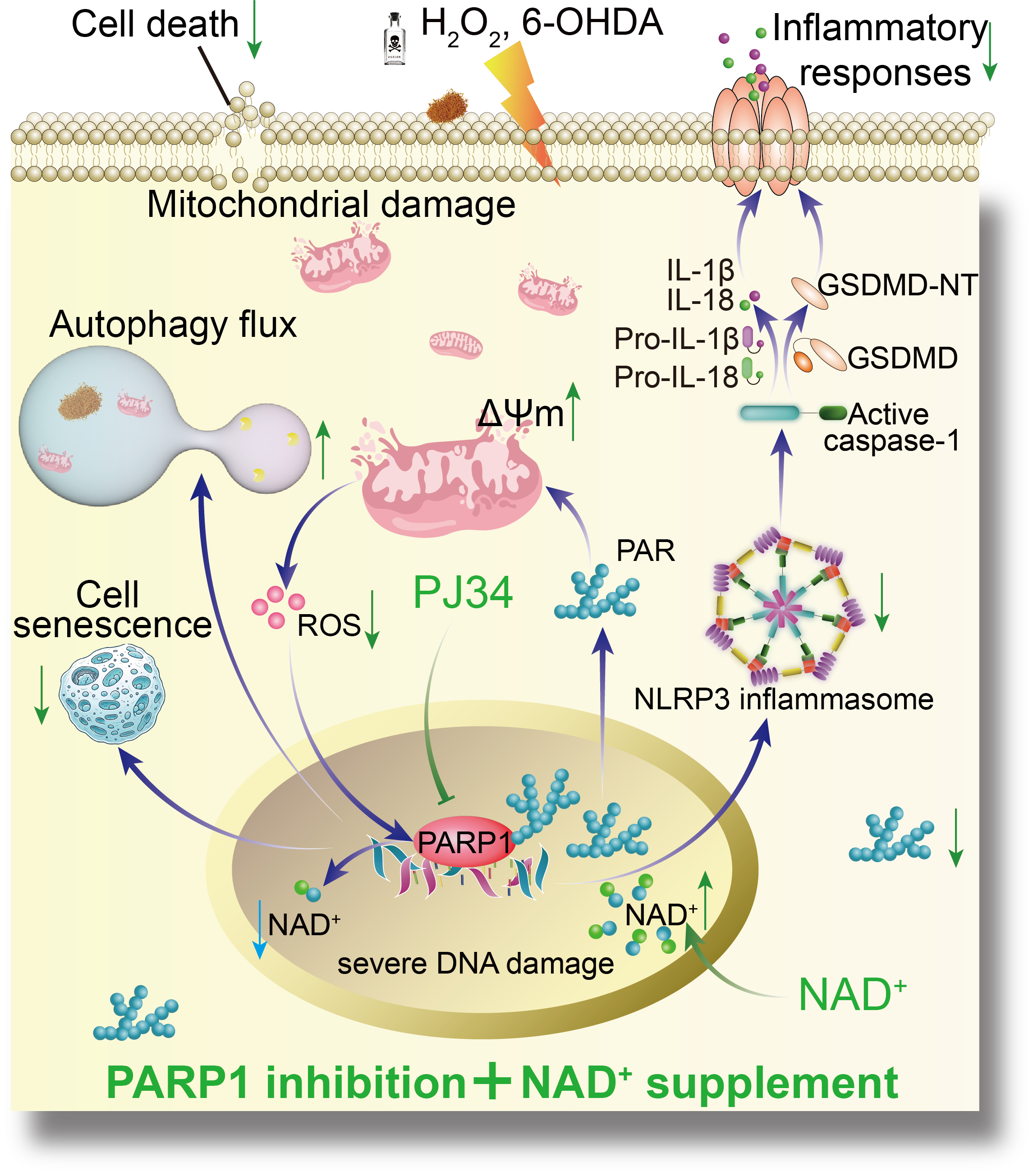


**Fig. S9.** The schematic diagram of neuroprotection by PJ34 and NAD^+^ supplementation in a 6-OHDA-induced PD model. Oxidative stress induced by 6-OHDA and H_2_O_2_ leads to mitochondrial damage, autophagy impairment, and cell senescence. This is marked by increased ROS production and NLRP3 inflammasome activation, which triggers inflammatory responses and subsequent cell death. Overactivation of PARP1 exacerbates DNA damage and depletes NAD^+^, further promoting oxidative stress and inflammation. However, the combination of PARP1 inhibition by PJ34 and NAD^+^ supplementation counters these deleterious effects. PJ34 reduces PARP1 activity, thereby limiting DNA damage and preserving NAD^+^ levels. This enhances autophagy and maintains mitochondrial integrity, reducing ROS production and NLRP3 inflammasome activation. Consequently, inflammation and cell senescence are mitigated, providing neuroprotection in PD.


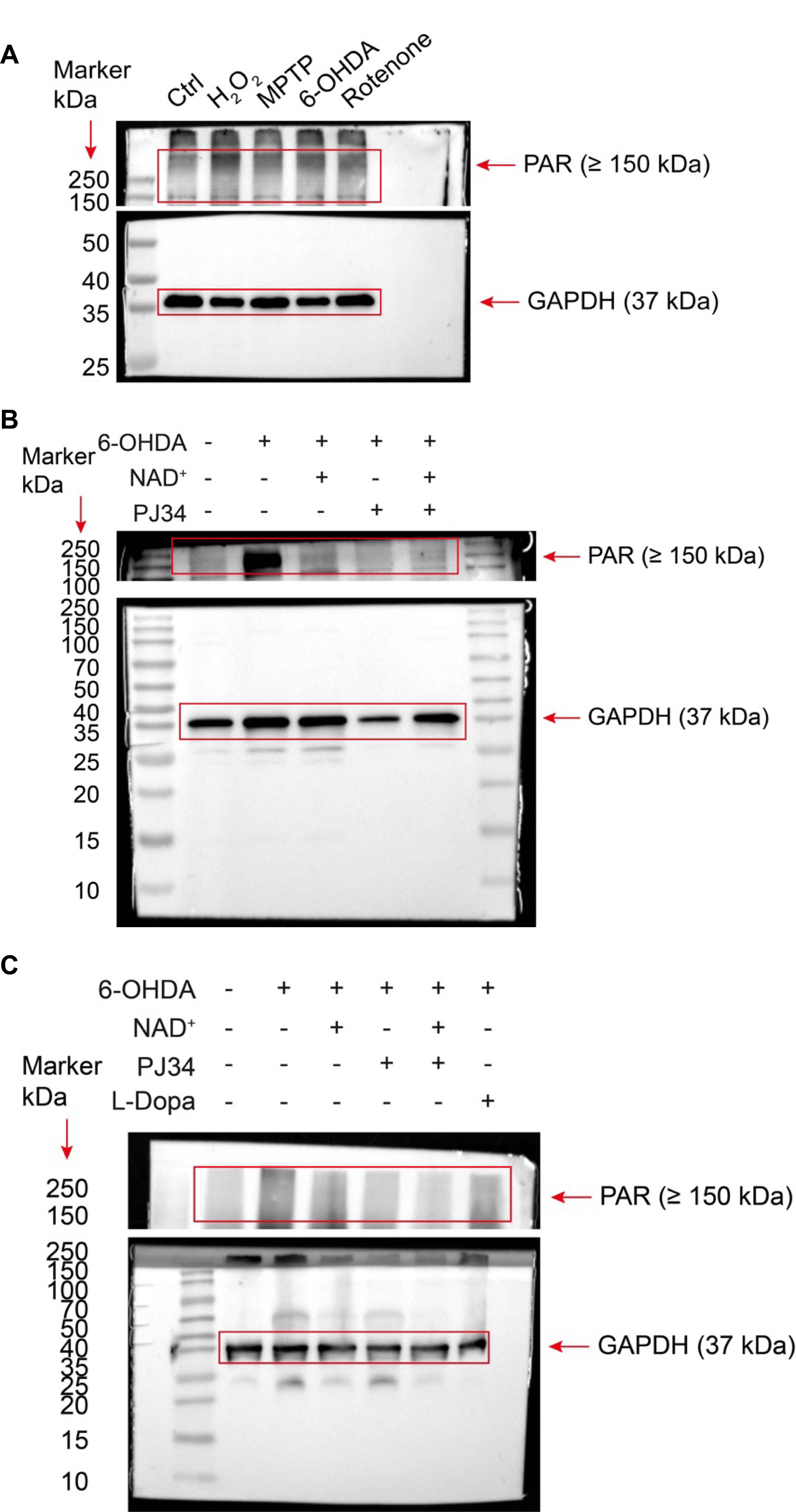


**Fig. S10** Original and non-processed Western blot images of Fig.S2C, S2N, and S8E.

**
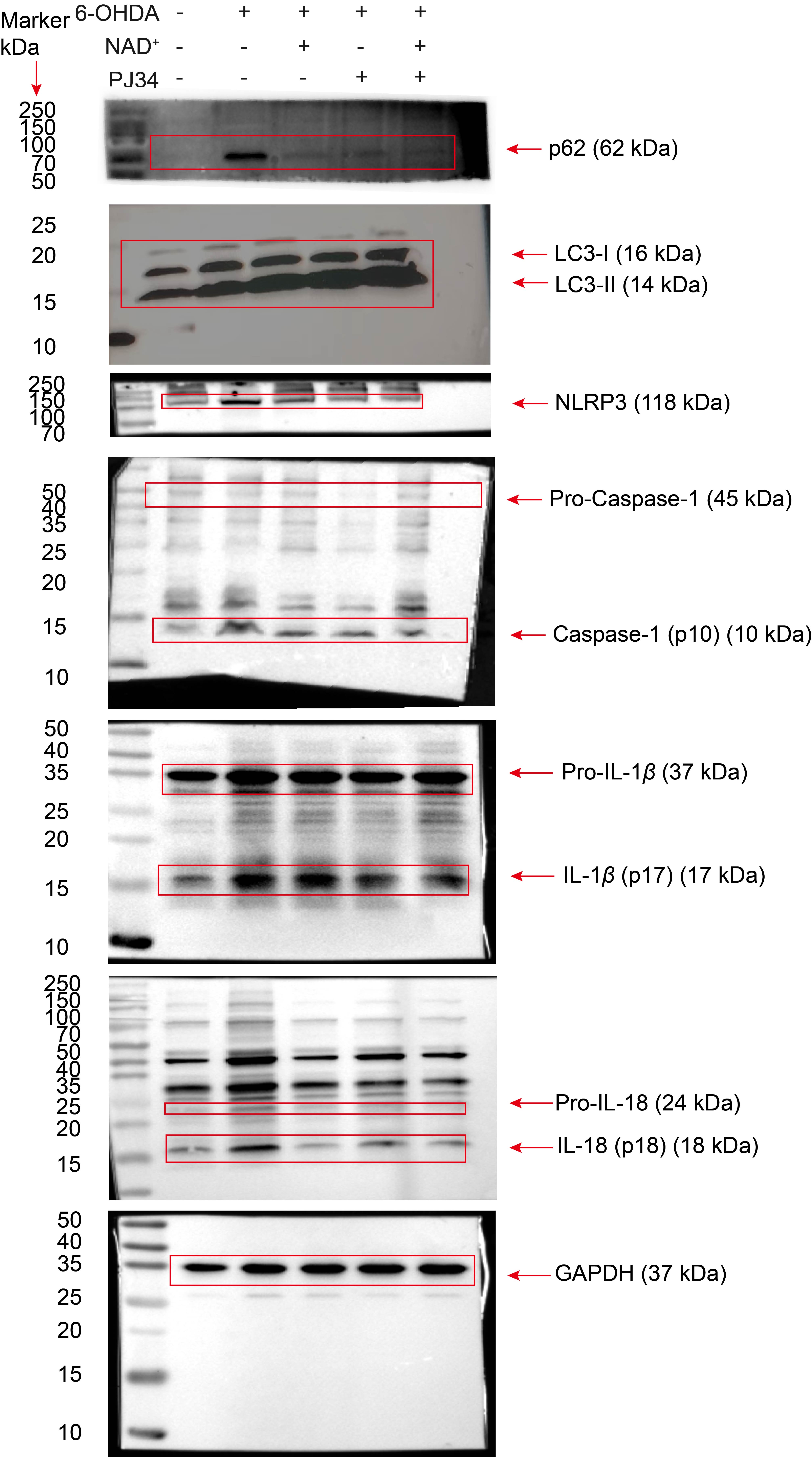
**

**Fig. S11** Original and non-processed Western blot images of Fig. 1C
